# Screening of Indigenous *Hanseniaspora* Strains from China for Ethanol Reduction in Wine

**DOI:** 10.3390/foods14071113

**Published:** 2025-03-24

**Authors:** Huimin Yang, Yue Wei, Wenqian Feng, Haoran Zhang, Jiao Jiang, Yi Qin

**Affiliations:** 1 College of Enology, Northwest A&F University, Yangling 712100, China; hmyang0416@163.com (H.Y.); wy031619@163.com (Y.W.); fwq18729205092@163.com (W.F.); zywmsjb@163.com (H.Z.); 2 National Forestry and Grassland Administration Engineering Research Center for Viti-Viniculture, Yangling 712100, China

**Keywords:** indigenous non-*Saccharomyces*, low-ethanol yield, ARTP, *Hanseniaspora*

## Abstract

Non-*Saccharomyces* yeasts have the potential to ameliorate wine ethanol levels, but such fit-for-purpose yeast strains are still lacking. Seventy-one indigenous non-*Saccharomyces* yeasts isolated from spontaneous fermentations of four wine regions in China (Ningxia, Xinjiang, Gansu, and Shaanxi) were screened for ethanol formation and were characterized for major metabolite profiles in synthetic grape juice fermentation to obtain non-*Saccharomyces* yeasts with low ethanol yields. Four *Hanseniaspora* strains with less volatile acidity production were primarily selected, and their ethanol yield was reduced by 22–32% compared to *S. cerevisiae*. These strains were further evaluated for oenological properties, namely ethanol and temperature tolerance, H_2_S production, and killer activities against *S. cerevisiae*. Strain HuC-3-2 was then subjected to Atmospheric Room Temperature Plasma (ARTP) mutagenesis, and a mutant (HuC32-2-72) with rapid growth and optimized ethanol-reducing capability was obtained. The best-performing strains were further characterized in sequential fermentations with *S. cerevisiae* in Merlot juice, and resulted in a 1.4% *v*/*v* decrease in ethanol yield. Comprehensive analysis of yeast populations and the production of key metabolites highlighted important carbon sinks, as well as glycerol formation, partially accounting for the ethanol reduction. In addition to ethanol amelioration, the *Hanseniaspora* strains also led to alterations in many metabolites, including volatile compounds and some organic acids, which can further modulate wine aroma and flavor.

## 1. Introduction

Due to climate warming and advances in viticultural management, wine regions located in warm viticulture areas, including the northwest of China, are commonly related to elevated sugar concentrations in ripe grape berries [1]. Consequently, ethanol levels in commercial wines have been progressively increased [2]. Excessive ethanol content in wines exerts difficulties on both alcoholic and malolactic fermentations by inhibiting the growth of yeast and lactic acid bacteria, thereby resulting in stuck and sluggish fermentation [3]. Inferior sensory qualities of such wines can also be observed due to increased perceptions of hotness, bitterness, astringency, and roughness, as well as the masking of some important aroma compounds by ethanol [4]. Furthermore, many consumers are becoming more health-conscious and mindful of their alcohol intake [5]. According to the International Wine and Spirits Record (IWSR), globally, the growth rate for the wines—no- or low-alcohol (NOLO) category has been well above that of the total wine market. Between 2015 and 2020, the average annual growth rate was 25 percent. The growth rate forecast by the IWSR from 2021 to 2025 is 15% per annum on average, compared with < 1% per annum for total wine volume [6]. Low-alcohol wines have become an important trend in the wine industry, with most countries following the same broad trend of replacing alcohol with low-alcohol alternatives. Thus, there is a strong demand to seek strategies to efficiently reduce ethanol levels in these wines.

To manage the ethanol content in wines, different approaches targeting all stages of winemaking have been proposed. Previous studies have indicated that carbonic maceration processing can be used to obtain a wine fraction with lower alcoholic strength [7]. *Pied de cuve* (PdC) technology, which refers to a method of indirect inoculation through an inoculum made from must that is already fermenting, has been confirmed to effectively control the production of alcohol and the interaction between a low-ethanol environment and the population dynamics of non-*Saccharomyces* yeast [8]. The method of reducing the alcohol content in wine by industrially evaporating ethanol and then mixing it has a smaller impact on sensory characteristics [9]. Furthermore, the inhibitor MKS1 regulates the reverse reaction of yeast metabolism to increase glycerol and reduce ethanol [10]. Among these strategies, screening lower-ethanol-producing non-*Saccharomyces* yeasts has attracted more attention [11]. A few strains of *Candida* spp. [12], *Hanseniaspora* spp. [13], *Lachancea* spp. [14], *Metschnikowia* spp. [15], *Zygosaccharomyces* spp. [16], and *Starmerella* spp. [16], showed 0.3–3.8% *v*/*v* less ethanol formation compared to that of *Saccharomyces cerevisiae* during wine fermentation. Despite their potential, fit-for-purpose non-*Saccharomyces* strains are still lacking. These oenological characteristics can be diminished under vinification conditions and often require a combination with a more robust starter culture, such as commercial *S. cerevisiae,* to mitigate the risk of slow and sluggish fermentation [17]. During mixed fermentation, the rapid growth and strong fermentation capability of non-*Saccharomyces* are required upon fermentation onset. To improve the yeast growth rate, different strain optimization strategies, such as mutagenesis, hybridization, and adaptive laboratory evolution, have been widely used [18,19]. The technology of Atmospheric Room Temperature Plasma (ARTP) is a novel and powerful mutagenesis tool using radio-frequency atmospheric pressure glow discharge plasma jets. Compared to conventional mutagenesis techniques, ARTP offers a more efficient approach to inducing DNA damage and producing stable mutant strains [20]. Because of its operational simplicity, cost-effectiveness, and avoidance of hazardous chemicals, ARTP has been extensively utilized in the mutagenesis of bacteria, fungi, and microalgae, demonstrating significant potential for enhancing productivity and optimizing desirable traits [21,22]. It is, therefore, hypothesized that ARTP can be utilized to enhance the growth rate of lower-ethanol-producing yeasts.

The present work aimed to generate and characterize lower-ethanol-producing yeast strains with rapid growth. Four low-ethanol-producing *Hanseniaspora* strains were first screened from 71 indigenous non-*Saccharomyces* strains based on ethanol yield and fermentation traits. One of the strains, HuC-3-2, was then subjected to ARTP mutagenesis to allow the generation of a novel low-ethanol-formation strain that is capable of rapidly initiating alcoholic fermentation. The best-performing strains were further characterized in a sequential culture with *S. cerevisiae* in Merlot juice, to determine their fermentation performance, with a focus on ethanol levels and the alterations of the primary and secondary metabolites.

## 2. Materials and Methods

### 2.1. Yeast Strains and Culture Conditions

The 71 indigenous non-*Saccharomyces* yeast strains used in this study were isolated from spontaneous fermentations using grapes sourced from four wine-producing regions of China, namely Ningxia, Xinjiang, Gansu, and Shaanxi. These yeast isolates were preserved in 20% glycerol stocks at −80 °C, and are listed in Table 1. The cryogenically preserved strains were revived for 2 days at 28 °C on YPD plates (2% glucose, 2% peptone, 1% yeast extract, 2% agar). Yeast starter cultures were prepared by inoculating approximately 5 × 10^6^ cells/mL into 250 mL Erlenmeyer flasks containing 100 mL YPD liquid medium, and incubated overnight at 28 °C with agitation at 150 rpm.

The commercial yeast *S. cerevisiae* CECA (Angel Yeast, Yichang, China) was used as the reference strain.

### 2.2. Screening of Strains with Low Ethanol Production

Lab-scale alcoholic fermentation trials (150 mL) were performed to screen for indigenous yeast strains with low ethanol yields. Fermentations were conducted in triplicate in a chemically defined medium (‘Triple M’ synthetic grape juice, 100 g/L glucose, 100 g/L fructose) at 20 °C [23]. Overnight yeast cultures were prepared in YPD liquid medium at 28 °C prior to being inoculated into Triple M at a rate of 1 × 10^6^ cells/mL. All tested strains were characterized for their ethanol yield and the change in ethanol yield, which were calculated by the following formula.

Ethanol yield (g/g) = ethanol production (g/L)/sugar consumption (g/L)(1)

Reduction in ethanol (%) = (A − B)/B × 100%(2)

where A refers to the ethanol yield of the tested non-*Saccharomyces* strain, whilst B is the ethanol yield of CECA at the same sugar consumption.

Strains with sugar consumption above 170 g/L and ethanol yield below 0.32 g/g (approximate 20% ethanol reduction compared with CECA) were selected from all tested indigenous non*-Saccharomyces* strains.

### 2.3. Evaluation of Enological Performance of the Candidate Strains

*Candidate* strains with low ethanol yields from the above screening assay were subjected to characterization for their oenological properties. Based on previous research findings, we conducted an ethanol tolerance assay within a 150 mL liquid YPD medium, with ethanol concentrations ranging from 2% to 8% (*v*/*v*), incremented by 2% (*v*/*v*), the initial yeast cell concentration at 1 × 10^6^ cells/mL, and shaking at 150 rpm. Yeast culture density was monitored 48 h after inoculation using a Cary 60 UV-Vis spectrophotometer (Agilent Technologies, Santa, Clara, CA, USA) at 600 nm. Similarly, yeast growth was characterized in normal YPD at four different temperatures (10, 20, 30, 40 °C). H_2_S production was evaluated using the BiGGY agar approach according to a previous study [24]. Killer toxin activity of the indigenous strains against the sensitive *S. cerevisiae* strain 1296, at 28 °C, was assessed using the agar diffusion well method, as previously described [25].

### 2.4. ARTP Mutagenesis to Generate Novel Yeast Strains with Lower Ethanol Yield

Atmospheric and Room Temperature Plasma (ARTP) mutagenesis was performed using the ARTP biological mutagenesis system (Wuxi Yuanqing Tianmu Biological Technology Co., Ltd., Wuxi, China). Mid-log phase non-*Saccharomyces* yeast cells were collected, washed twice with 5% PBS, and diluted to a suspension with an OD_600_ (the optical density value measured at a wavelength of 600 nm) of 0.6–0.8. A dose of 10 μL yeast culture was then evenly spread on a sterile metal plate, and was exposed to 100 W power of radio frequency for 0 -150 s. The culture was sampled at various time points during the ARTP treatment to determine the lethality rate, which was calculated as the ratio of the total number of dead spores after the ARTP treatment to that of the live spores before the treatment. Simultaneously, samples were collected and eluted with 1 mL sterile PBS (5%). Cells were then harvested after centrifugation (5000 rpm, 10 min), and the medium was replaced with YPD and sterile glycerol (added to 20% *v*/*v*) for cryo-storage.

### 2.5. Screening for the ARTP Mutagenesis Isolates with Lower Ethanol Yield via Micro- and Lab-Scale Fermentations

Glycerol stocks of the mixed culture collected at the mutagenic lethality of 80–90% were streaked onto YPD agar medium for isolation of single colonies. Preliminary characterization of these single colonies was performed in micro-scale fermentations in 24-well micro-titer plates. Each well containing 1.8 mL YPD was inoculated with approximately 1 × 10^6^ cells /mL yeast isolates. Plates sealed with breathable titer tops (Coad: SF-200) were incubated at 20 °C for 72 h, after which the cell density was measured using a microplate spectra-photometer (BioTek, ELx800, Winooski, VT, USA) at 600 nm. Mutants with higher biomass than the parent strain were selected for further evaluation by lab-scale sequential fermentations.

The filter-sterilized Merlot juice (2021, Yuma winery, Ningxia, China) with 254 g/L sugar was used for the lab-scale fermentation experiment. Sequential fermentations were performed in triplicate in 500 mL Erlenmeyer flasks equipped with air locks and containing 300 mL of grape juice at 25 °C. The Merlot juice was first inoculated with overnight cultures of the selected non-*Saccharomyces* at 1 × 10^7^ cells/mL, followed by inoculation of the commercial wine yeast CECA at 1 × 10^6^ cells/mL after 48 h. Fermentation samples were collected every 48 h to determine yeast viability and sugar consumption. The final sample was collected at the end of fermentation for downstream analyses of metabolites and volatile compounds.

### 2.6. Chemical Analysis

Residual sugar and volatile acidity were measured according to a previously described process [26]. Glycerol, ethanol, and organic acids were quantified using HPLC equipment (Agilent 1100, Agilent Technologies, Santa, Clara, CA, USA) equipped with an HPX-87H Aminex ionexchange column (300 mm × 7.8 mm, 9 µm particle size, 8% cross-linkage; Bio-Rad, Hercules, CA, USA). Specifically, citric, tartaric, pyruvic, malic, succinic, fumaric, and acetic acids were assayed by a diode array detector (Agilent DAD G7115A, Agilent Technologies, Santa, Clara, CA, USA) at 210 nm with the column temperature maintained at 60 °C whilst an extra cation H^+^ micro-guard cartridge (BioRad, Hercules, CA, USA) was included for the determination of glycerol and ethanol at 40°C. Prior to the HPLC analysis, samples were diluted 1 in 5 using ultra-pure water, and filtered through 0.22 μM nylon syringe filters. A volume of 20 μL of diluted samples was finally injected into the HPLC equipment. Analytes were eluted with 5 mM H_2_SO_4_ at a flow rate of 0.6 mL/min. Quantitative analusis of the analytes was performed using the standard curves.

Volatile compounds were analyzed using head space–solid phase microextraction–gas chromatography with mass spectrometry (HS-SPME-GC-MS) following Chen et al. [26]. In brief, an Agilent 6890 GC coupled with an Agilent 5975B MSD (Agilent Technologies, Santa, Clara, CA, USA) and an HP-INNOWAX (J&W Scientific, Folsom, CA, USA; 60 m × 0.25 mm, 0.25 μm film thickness) column were used for volatile profiling. The carrier gas was helium at 1 mL/min; the solid phase microextraction was manually injected by placing the SPME fiber at the GC inlet for 25 min in a non-split mode. The temperature of the GC system was adjusted as follows: 40 °C for 5 min, then increased to 200 °C at a rate of 3 °C/min and held for 2 min. The ion source temperature was kept at 230 °C. Spectra were acquired on electron ionization (EI) at 70 eV, and the mass scan range was 29–350 *m*/*z*, with a scan interval of 0.2 s.

Standard calibration curves were obtained using volatile compound standards in a synthetic wine medium (14% *v*/*v* ethanol, 5 g/L tartaric acid, pH 3.8). The standard mixture was blended with a 10 µL internal standard (4-methyl-2-pentanol, 20 mg/L), and analyzed according to the HS-SPME-GC-MS protocol described above. Agilent ChemStation was used to qualify and quantify the volatile compounds. The concentrations of the compounds were calculated using calibration curves following [26].

### 2.7. Statistical Analysis

Data were preliminarily processed using SPSS 26.0 (SPSS Inc., Chicago, IL, USA), and are expressed as mean values ± standard deviations. The same software was used to perform One-way ANOVA to allow the determination of significant differences among the tested strains. A confidence interval for One-way ANOVA was set at 95%. Volatile compounds with means that are significantly different (*p* < 0.05) across the treatments were further analyzed by principal component analysis (PCA) using Origin 2024 (Originlab Inc., Northampton, MA, USA). The rest of the figures were plotted using GraphPad Prism 9 (GraphPad Software, San Diego, CA, USA).

## 3. Results

### 3.1. Evaluation of Non-Saccharomyces Yeasts with Low Ethanol Yield

**Figure 1:**
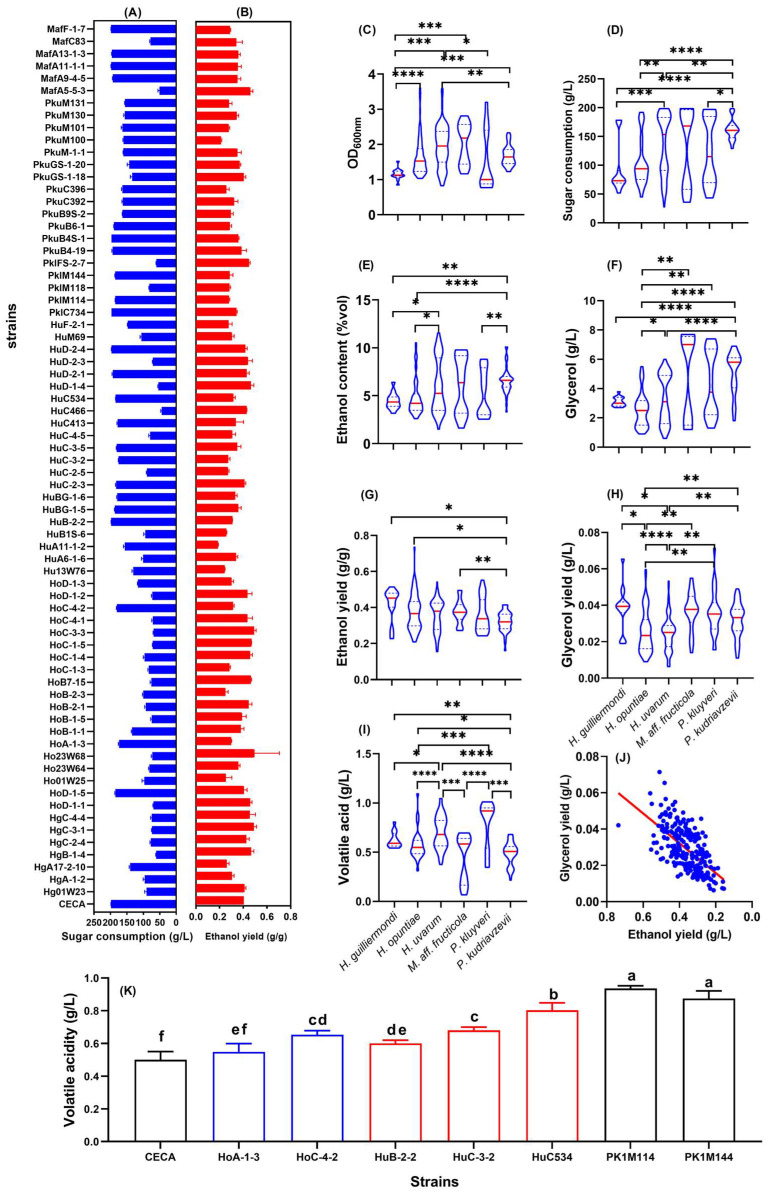
Physiological parameters of fermented Triple M medium by non-*Saccharomyces* yeasts. (**A**) Sugar consumption of each tested yeast strain; (**B**) ethanol yield of each tested yeast strain. Graphs (**C**–**G**) are violin plots demonstrating interspecific differences of the fermentation-related parameters. (**C**) Culture density at 600 nm; (**D**) sugar consumption; (**E**) ethanol content; (**F**) glycerol content (**G**) ethanol yield; (**H**) glycerol yield; (**I**) volatile acidity; (**J**) total acidity; (**K**) volatile acidity of 7 candidate non-Saccharomyces strains with low ethanol yields. * *p* < 0.05; ** *p* < 0.01, *** *p* < 0.001; **** *p* < 0.0001; Different letters indicate differences among wine samples determined by the Duncan test at a 95% confidence level.

The ethanol yields of 71 indigenous non-*Saccharomyces* strains and *S. cerevisiae* CECA were assessed by fermentation in Triple M with an initial sugar concentration of 200 g/L. The physicochemical parameters of all ferments were determined, mainly to evaluate the ability of the tested strains to induce and conduct alcoholic fermentation (Figure 1). The reference strain, CECA, consumed 198.18 g/L of sugar to produce 10.16% *v*/*v* ethanol, with an ethanol yield of 0.404 g/g. By contrast, the amount of sugar consumed by the tested non-*Saccharomyces* isolates ranged from 43.93 to 198.15g/L (Figure 1A), and their ethanol yield ranged from 0.290 to 0.494 g/g (Figure 1B). In order to select lower-ethanol-forming non-*Saccharomyces* yeasts with good fermentation performance, the ethanol yield of all tested strains was compared to that of CECA at the same sugar consumption level. Here, we proposed a basic selection criterion for such low-ethanol-yield non-*Saccharomyces* yeasts, which includes the capability to utilize a minimum amount of 170 g/L sugar whilst having an ethanol yield of less than 0.32 g/g. Accordingly, strains HoA-1-3, HoC-4-2, HuB-2-2, HuC-3-2, HuC534, PklM114, and PklM144 were identified as candidates with the potential for ethanol reduction. Compared to CECA, the mentioned strains displayed decreased ethanol yields by 26.03%, 22.33%, 23.77%, 32.05%, 21.49%, 30.64%, and 29.24%, respectively (Figure 1A). The reduction in ethanol yields indicated a higher conversion of sugar from ethanol formation through alternate metabolic pathways. In the present study, glycerol and volatile acidity were determined after the termination of alcoholic fermentation. These parameters were further clustered at the species level, along with ethanol yield, culture density, and sugar consumption, to reveal a general picture of interspecific differences in sugar metabolism (Figure 1C–J).

Strains belonging to *H. uvarum*, *P. kluyveri,* and *M. aff. fructicola* displayed better growth and more sugar consumption in Triple M compared to *H. guilliermondi*, *H. opuntiae,* and *P. kudriavzevii* strains (Figure 1C,D). With regard to major metabolites during alcoholic fermentation, significant differences were observed in ethanol and glycerol production among the six yeast species (Figure 1E–H). Particularly for *M. aff. fructicola* strains, the production of ethanol and glycerol fluctuated the most, which were within the range of 2.97–8.88% *v*/*v* and 1.37–7.60 g/L, respectively. Interestingly, the ethanol yields of the 71 non-*Saccharomyces* strains were negatively associated with glycerol production (Figure 1J). Volatile acidity (expressed as acetic acid) of the ferments by the end of fermentation was within the range of 0.37–0.85 g/L, among which *H. uvarum* and *P. kluyveri* resulted in a higher volatile acidity level compared to other species (Figure 1I). Notedly, the seven candidate strains with lower ethanol yields produced significantly higher amounts of volatile acidity compared to CECA, but all were nonetheless within the legal limit (1.2 g/L) (Figure 1K). Due to their lower volatile acidity yields, strains HoA-1-3, HoC-4-2, HuB-2-2, and HuC-3-2 were further shortlisted for subsequent characterization.

### 3.2. Oenological Properties of Low-Ethanol-Yielding Hanseniaspora Yeasts

All strains were able to tolerate an initial glucose concentration of 400 g/L (Table 2). However, a decrease in growth for *Hanseniaspora* strains was shown when the initial sugar concentration exceeded 300 g/L. The impacts of ethanol and temperature on the survival of the four selected *Hanseniaspora* strains were evaluated (Table 2). In general, increased ethanol concentration resulted in reduced yeast viability. Strains HoA-1-3 and HoC-4-2 displayed significantly reduced viability in medium with 6% *v*/*v* or more ethanol, whilst the growth density of HuB-2-2 and HuC-3-2 decreased remarkably only with 4% *v*/*v* or more ethanol (Table 2). In terms of temperature tolerance assays, growth was observed for all strains between 10 °C and 40 °C, among which the growth density was the highest for strains HoC-4-2 and HuB-2-2 at each tested temperature. Strains HoA-1-3 and HuC-3-2 showed considerable growth at typical wine fermentation temperatures (20 °C and 30 °C), whilst less population was seen at the two extreme temperatures (10 °C and 40 °C, Table 2).

Hydrogen sulfide production by the four strains was evaluated using the plate-based method by comparing the colors of the colonies grown on the BiGGY agar. HuB-2-2 was the only strain that is capable of producing H_2_S, which was indicated by the darker color of all triplicate colonies (Table 2). Further, no killer activity was observed for any of the four *Hanseniaspora* strains against the sensitive-type strain *S. cerevisiae* 1296 (Table 2).

**Figure 2:**
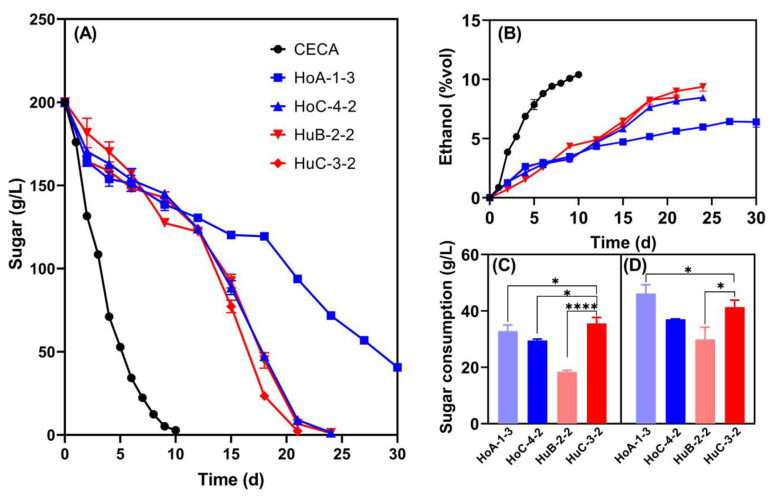
Fermentation performance of the four *Hanseniaspora* strains in Triple M. (**A**) Sugar consumption kinetics; (**B**) ethanol yield kinetics; (**C**) the amount of sugar consumed on the second day of the fermentation; (**D**) the amount of sugar consumed on the fourth day of the fermentation. * *p* < 0.05; **** *p* < 0.0001.

The sugar consumption and low-ethanol-production capacity of the four strains were double-checked in Triple M fermentations to ensure the stability and reliability of such traits (Figure 2). Undoubtedly, CECA showed the strongest ethanol production capacity, with 10.41% *v*/*v* ethanol being produced, which was significantly higher than that of the four *Hanseniaspora* strains. At the end of fermentation, the ethanol contents of HoA-1-3, HoC-4-2, HuB-2-2, and HuC-3-2 were 6.41% *v*/*v*, 8.47% *v*/*v*, 9.38% *v*/*v*, and 8.48% *v*/*v*, respectively (Figure 2B). Notedly, HoA-1-3 was unable to complete fermentation, whilst sugar was thoroughly depleted for the other three strains, but the fermentation duration doubled compared to the CECA monoculture fermentation (Figure 2A). Normally, *S. cerevisiae* is added into the ferments several days after the inoculation of non-*Saccharomyces* to ensure timely wine fermentations. Thus, the sugar consumption of the selected strains was further compared on the 2nd and the 4th day of fermentation (Figure 2C). HuC-3-2 consumed higher amounts of sugar compared to HoC-4-2 on the 2nd day, but no significant differences were observed on the 4th day (Figure 2C). Based on the above results, strain HuC-3-2 was selected as the parent strain for further reducing ethanol yield via ARTP mutagenesis.

### 3.3. ARTP Mutagenesis and Rapid Screening of Yeasts with Lower Ethanol Yield

**Figure 3:**
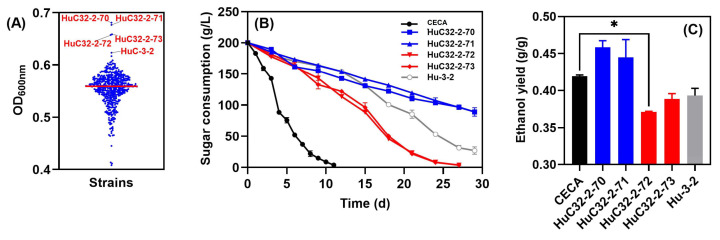
Evaluation of the 488 single isolates derived from ARTP mutagenesis. (**A**) Culture density at 600 nm after 72 h of growth in YPD; (**B**) sugar consumption kinetics of the four isolates with the greatest growth; (**C**) ethanol yield of the four candidate isolates. * *p* < 0.05.

With a view to improving growth whilst further reducing the ethanol yield of the indigenous non-*Saccharomyces* yeast, the strain HuC-3-2 was subjected to ARTP mutagenesis. Cell counts were conducted to determine yeast viability at intervals of 45 s, 55 s, and 65 s, in which the lethality rates of the treated cells reached 80~90%. A total number of 488 ARTP-mutagenized strains were obtained, and individual isolates were evaluated for their relative performance in micro-scale screening experiments. The basis for the preliminary selection of candidate strains was an improved biomass in comparison to the parent strain, HuC-3-2. Consequently, four strains, namely, HuC32-2-70 (OD_600_ = 0.681), HuC32-2-71 (OD_600_ = 0.677), HuC32-2-72 (OD_600_ = 0.659), and HuC32-2-73 (OD_600_ = 0.657), outcompeted HuC-3-2 (OD_600_ = 0.623) (Figure 3A), and were further evaluated in lab-scale fermentations in Triple M (Figure 3B). For CECA fermentation, sugar utilization was rapid, finishing in 12 days, whilst strains HuC32-2-72 and HuC32-2-73 consumed all sugar in 27 days. By contrast, fermentation was stalled for strains HuC32-2-70 and HuC32-2-71 (Figure 3B). Ethanol was analyzed by the end of fermentation, and the two mutants that competed for fermentation reduced ethanol yields markedly compared to those of CECA (Figure 3C).

### 3.4. Sequential Merlot Juice Fermentations Using Low-Ethanol-Yielding Hanseniaspora Yeasts and S. cerevisiae

#### 3.4.1. Dynamic Changes of Sugar Consumption and Yeast Population During Fermentation

**Figure 4:**
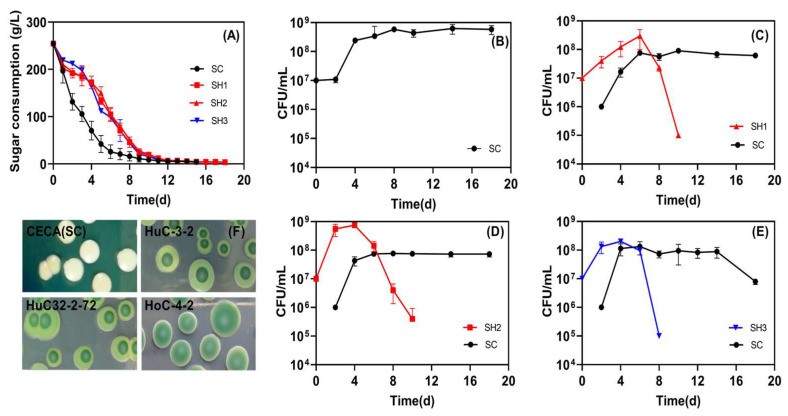
Sequential inoculation fermentation in Merlot grape juice. (**A**) Sugar consumption kinetics; (**B**) viability of CECA (SC) in pure culture fermentation; (**C**) yeast viability in SH1 fermentations (SH1: sequential inoculation fermentation of HuC-3-2 and CECA); (**D**) yeast viability in SH2 fermentations (SH2: sequential inoculation fermentation of HuC32-2-72 and CECA); (**E**) yeast viability in SH3 fermentations (SH3: sequential inoculation fermentation of HoC-4-2 and CECA); (**F**) yeast counts as determined on WLN agar plates during fermentation.

Three low-ethanol-producing isolates selected from this study, including one ARTP mutant, were evaluated for their potential application in winemaking in sequential fermentation with CECA (SH1, SH2, and SH3, respectively). The fermentation process was successfully completed for all respective treatments. Among them, the CECA monoculture fermentation exhibited the most rapid progression, whereas the sugar depletion in the mixed fermentations was observed to take place over a span of 18 days (Figure 4A), and the yeast population reached the stationary phase on Day 4, and retained over 10^8^ CFU/mL till fermentation terminated (Figure 4B). All tested non-*Saccharomyces* dominated at the early stage of mixed fermentations (4~6 d), followed by a sharp drop to less than 10^6^ CFU/mL on Day 10. Among them, the ARTP-mutagenized strain HuC32-2-72 had an OD value close to 10^9^ CFU/mL on Day 4, which was higher than HuC-3-2 and HoC-4-2 (Figure 4C–E). In all sequential fermentations, the population of CECA increased to approximately 10^8^ CFU/mL 2–4 days after inoculation, and the population was retained at this level afterward (Figure 4C–E).

#### 3.4.2. Basic Wine Parameters

The basic chemical components of pure and mixed fermented wines are listed in Table 3, many of which were impacted by the fermentation modalities. The ethanol concentrations in the sequential wines were approximately 1.4% *v*/*v* lower than the CECA wines, resulting in a reduced ethanol yield change rate of 10.42%. The sequential wines had higher glycerol concentrations than the control, most noticeably in SH2, which exhibited a significant increase of 46.1% in comparison to CECA. Furthermore, total acid concentrations, as well as malic and succinic acids, were significantly lower in the sequential wines compared to the control. A notably low pyruvic acid concentration was identified in SH2, recording decrements of 37.4%, 25.3%, and 32.7% relative to CECA, SH1, and SH3, respectively. However, the quantities of lactic and fumaric acids displayed negligible fluctuations across all wines. Compared to CECA monoculture wines, a higher amount of acetic acid was found in all sequential wines, which were nonetheless within the limit of the international standard (1.2 g/L, OIV, 2018).

#### 3.4.3. Volatile Compounds of the Wines

A total number of 40 volatile compounds were identified and quantified in Merlot wines, of which 37 showed significant differences between the sequential and CECA wines (ANVOA; *p* < 0.05, Table 4).

The impact of alcohol, which is a secondary metabolite generated by yeast during the process of alcoholic fermentation, on the aroma of wine can be either beneficial or detrimental [27]. Excessive contents of higher alcohols can negatively affect wine flavor [28]. Sequential inoculation caused a decrease in total higher alcohols, mainly driven by isobutanol, which was 1.4~2-fold less present than in CECA wines. The production of 2-methyl-1-propanol, 3-methyl-1-butanol, and phenylethyl alcohol during fermentation occurs via the conversion of valine, leucine, and phenylalanine via the Ehrlich pathway during fermentation [29]. Compared with CECA wines, 3-methyl-1-butanol and 2,3-butanediol were also significantly lower in SH1 and SH2 wines, but the amounts were comparable in SH3 wines. Phenethyl alcohol is a significant compound in wine, possessing a rose-honey flavor. It is produced by yeasts through the anaerobic conversion of phenylalanine [30]. In this study, phenylethyl alcohol was the only identified higher alcohol found to be higher in sequential wines than in the CECA wines, with the highest amount being seen in SH3 wines (Table 4). Farnesol and acetoin were remarkably increased in sequential wines, whilst nerol was found to be higher in the control wines than any of the sequential wines (Table 4).

Esters are formed mainly during alcoholic fermentation and are an important component of a wine’s aroma [31]. Generally, esters positively contribute to wine aroma and are considered major contributors to sweet and fruity odors [32]. In contrast to higher alcohols, in sequential wines, the acetate esters were generally higher than in the CECA wines, of which ethyl acetate was the most abundant, and it was 2~4-fold higher than in the control wines (Table 4). Isoamyl phenethyl acetates also surpassed their olfactory thresholds, and were 1.5~2-fold and 2.3~3.4-fold higher in the sequential wines, respectively (Table 4). Conversely, sequential inoculation resulted in a decrease in concentrations of most ethyl esters, particularly ethyl lactate and ethyl decanoate (Table 4). Non-*Saccharomyces* yeasts influence the production of ethyl esters in fruit wine, potentially due to the presence of secretases typically absent in *S. cerevisiae*, such as α-L-arabinofuranosidase, β-glucosidase, polygalacturonase, cellulase, and protease [33,34]. In sequential wines, the identified fatty acids were generally lower than in the CECA wines, except for octanoic and decanoic acids, which were either comparable or higher than the control (Table 4). A previous study reported that fatty acids have little to do with wine quality but play an essential role in the complexity of wine aromas [27,35]. The formation of terpenes was also affected by the fermentation matrix.

**Figure 5:**
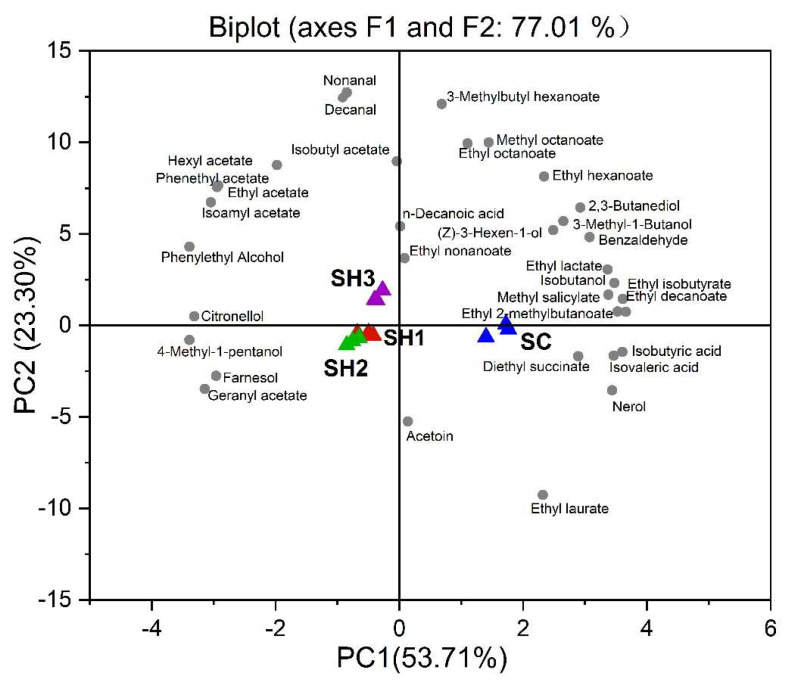
Principal component analysis of the volatile profiles in Merlot wines. Yeast isolates (treatments) are presented using colored triangles, and the volatile compounds in grey dots. SC (CECA): *S. cerevisiae*, SH1: sequential inoculation fermentation of HuC-3-2 and CECA, SH2: sequential inoculation fermentation of HuC32-2-72 and CECA, SH3: sequential inoculation fermentation of HoC-4-2 and CECA.

Principal component analysis (PCA) was further performed to visualize the discrimination of the entire set of volatile data of the resultant wines (Figure 5). The first two principal components (PCs) clearly separated the Merlot wines made with different fermentation modalities, and accounted for 77.01% of the variance in data (Figure 5). The first PC (F1:53.71%) separated the wine samples between sequential wines and the CECA wines, with SH1, SH2, and SH3 on the left-hand side of the plot, and SC on the right-hand side of the plot. The compounds that appear to be driving the separation along PC1 were isobutyric acid, isovaleric acid, n-decanoic acid, some ethyl esters, and many higher alcohols (Figure 5). Mixed inoculation treatments were associated with the production of acetate esters, phenylethyl alcohol, and some terpenes. The SH3 wines were separated along PC2 (higher left quadrant, Figure 5) from the remaining two mixed-inoculation wines. The SH3 wines were characterized by higher amounts of major acetate esters and phenylethyl alcohol, whereas the SH1 and SH2 wines were located at the negative axis of PC2, and were associated with 4-methyl-1-pentanol, farnesol, geranyl acetate, and acetoin (Figure 5).

## 4. Discussion

Yeast strains that are capable of reducing ethanol levels in wine whilst enhancing the overall wine quality are highly demanded by winemakers. In this study, four low-ethanol-producing strains, *H. uvarum* HuC-3-2 and HuB-2-2, and *H. opuntiae* HoC-4-2 and HoA-1-3, were primarily selected from 71 indigenous non-*Saccharomyces* strains obtained from four wine-producing regions in China (Figure 1B and Table A1). These four *Hanseniaspora* strains required more than 24 g/L of consumed sugar to produce 1% *v*/*v* of ethanol in Triple M medium (Table A2). Contrarily, it is documented that *S. cerevisiae* yeast uses 16.83 g/L to 17 g/L on average [36]. *Hanseniaspora* has been reported as one of the predominant yeast species present during the early stages of wine fermentation, of which many strains were able to lower wine ethanol [37,38]. The ethanol yield of *Hanseniaspora* spp. exhibited both interspecies and intraspecies variability, as shown in Figure 1. In this study, the ethanol yields of *H. uvarum* and *H. opuntiae* were found to be 0.185–0.464 g/g and 0.250–0.492 g/g, respectively (Table A2). There is also a finding that the ethanol yield from the isolates of *H. uvarum* and *H. opuntiae*, collected from South African grape must and vineyards, ranged between 0.28 and 0.53 g/g and 0.44 and 0.54 g/g, respectively [39].

In addition to the superiority in reducing ethanol yields of the four *Hanseniaspora* strains, it is also important to highlight that both strains completed fermentation in a medium with 200 g/L sugar and produced 7–9% *v*/*v* ethanol (Table A2). This indicated their better tolerance to higher concentrations of ethanol during fermentation, whilst cell growth was observed in the presence of 8% *v*/*v* ethanol (Table 2). A higher tolerance to ethanol would be beneficial for glucose uptake and ensure normal fermentation rates under oenological conditions since ethanol alters cell membrane structure [40]. Apart from ethanol tolerance, the four *Hanseniaspora* strains can also resist low fermentation temperatures (Table 2), indicating their potential applicability in producing low-ethanol white wines with desirable aroma profiles [41]. Additionally, three strains (except HuB-2-2) did not produce H_2_S, which has a negative organoleptic impact in wines [42,43], nor did they present any killer activities against *S. cerevisiae* (Table 2).

Strain HuC-3-2 outcompeted the other three strains due to its shorter fermentation duration and greater sugar consumption during the first two days in the Triple M medium (Figure 2). This strain was further subjected to ARTP mutagenesis, and a mutant strain, HuC32-2-72, with both low-ethanol-producing capability and rapid growth, was finally selected (Figure 3). ARTP, as a newly developed mutagenesis system, has been widely utilized in microorganism breeding. In addition to being environmentally friendly and easy to operate, ARTP is more convenient and effective in obtaining a higher mutation rate of microbes due to its stronger DNA damage capacity than traditional methods [42]. The mutant strains derived via ARTP mutagenesis are more able to obtain the target phenotypes, e.g., having an improved specific growth rate than the wild-type strains [21]. Nonetheless, there is a lack of studies on utilizing ARTP to reduce the ethanol yields of yeast strains. To the best of our knowledge, we reported the first successful case of using ARTP mutagenesis in wine yeast for more rapid growth whilst further reducing its ethanol yield (Figure 3).

The low-ethanol-producing phenotype of HuC-3-2, HuC32-2-72, and HoC-4-2 was then validated in a series of mixed inoculation treatments in Merlot juice with a higher initial sugar concentration, where ethanol modulation becomes further relevant. Fermentations inoculated with *S. cerevisiae* CECA alone finished first (Figure 4A), whereas the addition of *S. cerevisiae* induced a rapid population decline in *Hanseniaspora* (Figure 4C–E). Such a decrease might be attributed to the interaction between *S. cerevisiae* and *Hanseniaspora*, and the inhibitory impact of the multi-stressor wine environment, in particular, ethanol stress and the competition for nutrients [43]. The three selected *Hanseniaspora* strains allowed for an ethanol reduction of 1.4% *v*/*v* compared to *S. cerevisiae* CECA (Table 3). For *Saccharomyces* spp., alcoholic fermentation is usually efficient, with comparable ethanol yield between strains.

Strains belonging to *Hanseniaspora* have been reported to be able to reduce wine ethanol by 0.6–2.5% *v*/*v* when co-inoculated with *S. cerevisiae* [38]. In this study, sequential fermentations of *H. uvarum* HuC-3-2, *H. uvarum* HuC32-2-72, and *H. opuntiae* HoC-4-2 with *S. cerevisiae* in Merlot juice consistently resulted in a 1.4% *v*/*v* decrease in ethanol yield. The fate of carbon transferred during the fermentation of low-ethanol-producing non*-Saccharomyces* strains remains largely elusive. A decreased ethanol yield can be due to the respiratory metabolism in aerobic cultures [44]. In anaerobiosis, non-*Saccharomyces* yeast can transfer the carbon source in sugar metabolism to the non-ethanol metabolism terminal and lead to the formation of secondary metabolites, such as glycerol, organic acids, higher alcohols, and esters [15,16,45,46], or sinks that remain undetected. In the present study, we observed that *H. uvarum* HuC-3-2, *H. uvarum* HuC32-2-72, and *H. opuntiae* HoC-4-2 resulted in a decrease in ethanol content along with an increase in glycerol in both Triple M and Merlot juice fermentations (Figure 1J, Table 3). Similar results were reported before in wine fermentations [15], but our finding disagrees with others [47]. Our results indicated that glycerol yield, like other fermentation features, was an interspecies and strain-related trait.

Higher contents of acetic and lactic acids were found in sequential wines (Table 3), which was in agreement with previous studies [46,47]. In this study, a significant decrease in ethanol production was observed in mixed fermentations, even without any intervention on the oxygen level during fermentation, and without a detrimental increase in acetic acid (Table 3). Acetic acid is produced from the oxidation of acetaldehyde by aldehyde dehydrogenase or the hydrolysis of acetyl-CoA derived from pyruvate [48]. However, the sequential wines contained less pyruvic acid than the CECA wines (Table 3). Previous studies have also observed that the reduction in the ethanol yield by some non-*Saccharomyces* strains could not be fully explained by the overproduction of glycerol or organic acids, suggesting that respiration would be responsible, at least in part, for the decreased ethanol yield observed for these strains [49,50]. Sugars were probably partially consumed through the oxidative pathway to produce biomass and other unknown products.

Significant increases in acetate esters and ethyl esters in the sequential wines (Table 4) agree with a number of previous studies [47,51,52]. In general, esters produced during fermentation were dependent on the rate of ester synthesis and hydrolysis [53] In fact, esters remain the important carbon sink in a range of lower-ethanol yeasts, and have either negligible or positive sensory contributions. C_2_-alcohol O-acyl-transferases (AATs), which are encoded by *ATF1* and *ATF2*, are critical and versatile enzymes that can utilize alcohols and acyl-CoA to form acetate esters [54]. A previous study suggested that overexpression of *ATF1* enhanced the total ethyl acetate whilst decreasing the yield of ethanol slightly [55]. All of these findings highlighted the need for further research on *ATF1* expression and AATs activity by *Hanseniaspora*, so as to offer a partial explanation for ethanol decrease. Of particular interest was the 2-3-fold increase in ethyl acetate in the sequential wines compared to the CECA wines (Table 4), which was close to or surpassed the level at which ethyl acetate could impart spoilage to the resultant wines. Thus, the influence of elevated ethyl acetate on the olfactory quality of the sequential wines requires further investigation. In addition, our study also demonstrated that *Hanseniaspora* can vigorously produce several key aromatic compounds (phenylethyl acetate and isoamyl acetate) in mixed inoculation fermentation with *S. cerevisiae*, which is in accord with some previous studies [51,56].

## 5. Conclusions

This study described the selection, optimization (via ARTP mutagenesis), and comprehensive characterization of three *Hanseniaspora* strains for their potential use in low-ethanol-producing starter cultures. The characterization of oenological properties indicated that the *Hanseniaspora* strains might be suitable for initiating fermentation without the risk of producing off-flavors (e.g., H_2_S). The three strains finally obtained resulted in approximately 1.4% *v*/*v* less ethanol in mixed fermentation wines than the *S. cerevisiae* monoculture wines. The evaluation of the yeast population and yeast metabolites highlighted glycerol and some esters as potential carbon sinks, partially accounting for the ethanol reduction. Further analysis on volatile compounds in the wine suggested that these strains have promise for use in combination with *S. cerevisiae* to manage ethanol levels and modulate aromas in wine, laying the groundwork for their broader application. Considering that these positive benefits were observed in sterile laboratory-scale fermentations, future studies on industrial-scale fermentation and sensory tasting should be undertaken to assess the effects of *Hanseniaspora* strains on the flavor profiles of the wines. Furthermore, optimizing the fermentation parameters, such as yeast cell concentration, fermentation time, or temperature, among others, of *Hanseniaspora* strains in fermentation will be essential, because these yeasts might be used in the production of wines that will be appreciated by the average consumer.

## Figures and Tables

**Table 1:** Yeast strains used in this study.

Species	Strains	Geographic Origin
*Saccharomyces cerevisiae*	CECA (commercial wine yeast from Angel Yeast)	Ningxia
*Hanseniaspora guilliermondii*	Hg01W23, HgA12, HgB14, HgC24, HgC31, HgC44, HgD11, HgD15	Shaanxi
	HgA17210	Xinjiang
*Hanseniaspora opuntiae*	Ho01W25, Ho23W64, Ho23W68, HoA-1-3, HoB-1-1, HoB-1-5, HoB21, HoB23, HoB715, HoC13, HoC14, HoC15, HoC33, HoC41, HoC42, HoD12, HoD13	Shaanxi
*Hanseniaspora uvarum*	Hu13W76, HuBS6, HuB22, HuBG15, HuBG16, HuC23, HuC25, HuC01, HuC45	Shaanxi
	HuA616, HuA1112, HuM69	Xinjiang
	HuC413, HuC466, HuC534	Gansu
	HuF21	Ningxia
*Metschnikowia aff fructicola*	MafA553, MafA945, MafA1111, MafA1313	Xinjiang
	MafC83	Gansu
	MafF17	Ningxia
*Pichia kluyveri*	Pk_1_M114, Pk_1_M118, Pk_1_M144	Xinjiang
	Pk_1_C734	Gansu
	Pk_1_FS27	Ningxia
*Pichia kudriavzevii*	Pk_2_B419, Pk_2_B4S1, Pk_2_B61, Pk_2_B9S2	Shaanxi
	Pk_2_C392, Pk_2_C396	Gansu
	Pk_2_GS118, Pk_2_GS120, Pk_2_M11	Ningxia
	Pk_2_M100, Pk_2_M101, Pk_2_130, Pk_2_131	Xinjiang

**Table 2:** Oenological properties of the four *Hanseniaspora* yeasts with low ethanol yields.

Isolates	Sugar Tolerance Test (g/L)				Ethanol Tolerance Test (%)				Temperature Tolerance Test (°C)				H_2_S Production	Killer Phenotype
	100	200	300	400	2	4	6	8	10	20	30	40		
HoA13	+++	+++	+++	++	+++	+++	++	+	+	+++	+++	++	−	−
HoC42	+++	+++	+++	++	+++	+++	++	+	++	+++	+++	+++	−	−
HuB22	+++	+++	+++	++	+++	++	+	+	++	+++	+++	+	+	−
HuC01	+++	+++	+++	++	+++	+++	++	+	++	+++	+++	++	−	−

Notes: +++ = intensive response, ++ = moderate response, + = low response, − = no response.

**Table 3:** Concentrations of non-volatiles in Merlot wines produced with mixed *Hanseniaspora*/CECA and pure CECA fermentations.

	SC	SH1	SH2	SH3
Sugar (g/L)	4.36 ± 0.80 ^a^	4.02 ± 0.47 ^a^	3.61 ± 0.08 ^a^	3.75 ± 0.02 ^a^
Ethanol (% *v*/*v*)	15.13 ± 0.22 ^a^	13.74 ± 0.12 ^b^	13.76 ± 0.10 ^b^	13.75 ± 0.07 ^b^
Ethanol Yield (g/g)	0.48 ± 0.01 ^a^	0.43 ± 0 ^b^	0.43 ± 0 ^b^	0.43 ± 0 ^b^
∆ethanol (% *v*/*v*)	0	−10.42	−10.42	−10.42
Glycerol (g/L)	6.25 ± 0.08 ^a^	8.79 ± 0.09 ^b^	9.13 ± 0.07 ^bc^	8.93 ± 0.12 ^b^
Total Acidity (g/L)	8.18 ± 0.14 ^a^	7.80 ± 0.11 ^ab^	7.38 ± 0.28 ^b^	7.75 ± 0.23 ^ab^
Citric Acid (g/L)	0.15 ± 0 ^a^	0.14 ± 0.01 ^ab^	0.12 ± 0.02 ^b^	0.15 ± 0 ^a^
Tartaric Acid (g/L)	1.18 ± 0.04 ^c^	1.81 ± 0.09 ^a^	1.48 ± 0.14 ^b^	1.63 ± 0.08 ^ab^
Pyruvic Acid (mg/L)	59.89 ± 3.4 ^a^	50.20 ± 0.07 ^b^	37.49 ± 3.97 ^c^	55.72 ± 5.06 ^ab^
Malic Acid (g/L)	2.36 ± 0.20 ^a^	2.03 ± 0.04 ^bc^	1.81 ± 0.07 ^c^	2.11 ± 0.11 ^ab^
Succinic Acid (g/L)	3.58 ± 0.31 ^a^	2.79 ± 0.05 ^b^	2.87 ± 0.03 ^b^	2.87 ± 0.09 ^b^
Lactic Acid (g/L)	0.29 ± 0.01 ^a^	0.31 ± 0.03 ^a^	0.27 ± 0.04 ^a^	0.34 ± 0.09 ^a^
Fumaric Acid (g/L)	0.02 ± 0 ^a^	0.02 ± 0 ^a^	0.02 ± 0 ^a^	0.02 ± 0 ^a^
Acetic Acid (g/L)	0.54 ± 0.02 ^a^	0.66 ± 0.06 ^b^	0.60 ± 0.02 ^ab^	0.59 ± 0.03 ^a^

Notes: values are expressed with means ± standard deviations (n = 3). Different letters within rows indicate differences among wine samples determined by the Duncan test at a 95% confidence level. SC: pure fermentation of CECA; SH1: sequential inoculation fermentation of HuC01/CECA; SH2: sequential inoculation fermentation of HuC32-2-72/CECA; SH3: sequential inoculation fermentation of HoC42/CECA.

**Table 4:** Volatile compounds of the Merlot wines.

Compounds (mg/L)	SC	SH1	SH2	SH3	Odor Threshold (mg/L)	OVA	Odors
Isobutanol	164.12 ± 8.46 ^a^	87.01 ± 0.40 ^c^	91.05 ± 5.94 ^c^	114.31 ± 9.78 ^b^	40	>1	Fusel alcohol
4-Methyl-1-pentanol	13.20 ± 2.11 ^c^	43.27 ± 3.02 ^a^	35.63 ± 0.97 ^b^	32.60 ± 2.21 ^b^	1	<0.1	Almond, toasted
3-Methyl-1-Butanol	221.19 ± 1.51 ^a^	175.04 ± 0.26 ^c^	190.04 ± 9.31 ^b^	210.77 ± 5.84 ^a^	30	>1	whiskey, nail polish
1-Hexanol	4.40 ± 0.21 ^a^	4.22 ± 0.32 ^a^	4.07 ± 0.25 ^a^	4.65 ± 0.59 ^a^	8	0.1–1	Green, grass
(E)-3-Hexen-1-ol	0.20 ± 0.01 ^a^	0.18 ± 0 ^a^	0.17 ± 0 ^a^	0.21 ± 0 ^a^	1	0.1–1	Herbaceous, green
(Z)-3-Hexen-1-ol	0.20 ± 0.02 ^a^	0.17 ± 0 ^a b^	0.16 ± 0.01 ^b^	0.18 ± 0.02 ^a b^	0.4	0.1–1	Green, cypress
2,3-Butanediol	26.44 ± 3.75 ^a^	12.97 ± 0.57 ^b^	14.27 ± 1.11 ^b^	21.99 ± 3.46 ^a^	120	0.1–1	Butter, creamy
Phenylethyl alcohol	94.76 ± 0.31 ^c^	155.42 ± 10.55 ^b^	175.43 ± 3.12 ^a^	184.15 ± 3.26 ^a^	10	>1	Floral, rose
∑Higher alcohols	511.33 ± 8.32 ^b^	435.051 ± 10.79 ^d^	475.22 ± 9.50 ^c^	536.29 ± 8.84 ^a^			
Ethyl acetate	85.29 ± 7.31 ^d^	164.45 ± 7.33 ^c^	186.15 ± 2.69 ^b^	238.66 ± 13.68 ^a^	7.50	>1	Fruity, nail polish, balsamic
Isobutyl acetate	0.02 ± 0 ^a^	0.03 ± 0 ^b^	0.03 ± 0 ^b^	0.04 ± 0 ^b^	1.60	<0.1	Waxy, fruity, apple, banana
Isoamyl acetate	1.07 ± 0.02 ^d^	1.58 ± 0 ^c^	1.80 ± 0.05 ^b^	2.07 ± 0.02 ^a^	0.03	>1	Banana
Hexyl acetate	12.23 ± 1.12 ^b^	18.32 ± 3.39 ^ab^	16.55 ± 2.09 ^b^	23.91 ± 4.39 ^a^	0.67	<0.1	Fruity, floral
Geranyl acetate	11.25 ± 0.31 ^c^	12.70 ± 0.44 ^b^	13.74 ± 0.37 ^a^	12.47 ± 0.40 ^b^	0.06	0.1–1	Floral
Phenethyl acetate	0.19 ± 0 ^c^	0.49 ± 0.04 ^b^	0.45 ± 0.04 ^b^	0.65 ± 20.0 ^a^	0.25	>0.1	Honey, floral, fruity
∑Acetate esters	86.60 ± 0.73 ^d^	166.58 ± 7.37 ^c^	188.44 ± 2.76 ^b^	241.45 ± 13.65 ^a^			
Ethyl 2-methylbutanoate	24.21 ± 2.46 ^a^	2.18 ± 0.10 ^c^	6.09 ± 0.17 ^b^	8.61 ± 0.46 ^b^	0.02	>1	Apple, berry, sweet, cider, anise
Ethyl hexanoate	2.71 ± 0.39 ^a^	2.16 ± 0.02 ^b^	2.01 ± 0.04 ^b^	2.64 ± 0.02 ^a^	0	>1	Fruity, green apple, floral, violet
Ethyl lactate	3.16 ± 0.41 ^a^	1.69 ± 0.15 ^b^	1.70 ± 0.13 ^b^	2.20 ± 0.26 ^b^	146	<0.1	Fruity, buttery
Ethyl nonanoate	0.03 ± 0 ^b^	0.04 ± 0 ^a^	0.02 ± 0 ^c^	0.03 ± 0 ^b^	1.30	<0.1	Waxy, fruity, rose, rum
Diethyl succinate	0.86 ± 0.16 ^a^	0.62 ± 0.03 ^b^	0.60 ± 0.06 ^b^	0.59 ± 0.07 ^b^	6	0.1–1	Wine, fruity
Ethyl laurate	1.73 ± 0.12 ^a^	1.46 ± 0.08 ^b^	1.40 ± 0.07 ^b^	1.04 ± 0.02 ^c^	146	<0.1	Fruity, buttery
Ethyl hexadecanoate	0.94 ± 0.12 ^a^	0.93 ± 0.07 ^a^	0.92 ± 0.10 ^a^	0.86 ± 0.04 ^a^	1.50	0.1–1	Fatty, rancid, fruity, sweet
Ethyl decanoate	19.81 ± 2.94 ^a^	11.50 ± 0.74 ^b^	11.24 ± 1.47 ^b^	13.31 ± 0.15 ^b^	0.20	>1	Fruity, fatty
Ethyl octanoate	11.55 ± 0.24 ^b^	11.62 ± 0.23 ^b^	8.84 ± 0.22 ^c^	12.67 ± 0.30 ^a^	0	>1	Pear, apricot, fruity, pineapple
∑Ethyl esters	40.82 ± 3.19 ^a^	30.02 ± 0.62 ^bc^	26.73 ± 1.94 ^c^	33.36 ± 0.53 ^b^			
Isobutyric acid	16.83 ± 1.60 ^a^	6.34 ± 0.28 ^b^	6.66 ± 0.39 ^b^	6.93 ± 0.41 ^b^	2.30	>1	Cheese, butter, rancid
Isovaleric acid	1.83 ± 0.13 ^a^	1.15 ± 0.08 ^b^	1.22 ± 0.03 ^b^	1.22 ± 0.06 ^b^	0.03	>1	Fatty, sweet, cheese
Hexanoic acid	2.75 ± 0.37 ^a^	2.51 ± 0.12 ^a^	2.71 ± 0.16 ^a^	2.53 ± 0.05 ^a^	3.00	>1	Leafy, wood, varnish
Octanoic acid	1.34 ± 0.21 ^a^	1.44 ± 0.16 ^a^	1.60 ± 0.10 ^a^	1.44 ± 0.04 ^a^	0.50	>1	Butter, almond
n-Decanoic acid	0.99 ± 0.05 ^ab^	0.81 ± 0.16 ^b^	1.04 ± 0.04 ^a^	1.10 ± 0.04 ^a^	1	>0.1	Fatty, unpleasant
∑Fatty acids	22.75 ± 2.30 ^a^	11.44 ± 0.42 ^b^	12.18 ± 0.64 ^b^	12.12 ± 0.44 ^b^			
Nerol	0.22 ± 0 ^a^	0.044 ± 0 ^c^	0.08 ± 0 ^b^	0.05 ± 0 ^c^	0.5	0.1–1	Violets, floral
Farnesol	0.13 ± 0.01 ^c^	0.18 ± 0.02 ^b^	0.23 ± 0.01 ^a^	0.18 ± 0.01 ^b^	0.02	>1	Lemon, floral, anise, honey
Acetoin	11.10 ± 0.34 ^b^	39.43 ± 0.33 ^a^	11.93 ± 0.34 ^c^	12.39 ± 0.47 ^d^	150	0.1–1	Butter, cream
∑Terpenes	11.46 ± 0.33 ^b^	39.66 ± 0.31 ^a^	12.27 ± 0.33 ^c^	12.63 ± 0.45 ^d^			

Notes: values are expressed with means ± standard deviations (n = 3). Different letters within rows indicate differences among wine samples determined by the Duncan test at a 95% confidence level. SC: pure fermentation of CECA; SH1: sequential inoculation fermentation of HuC01/CECA; SH2: sequential inoculation fermentation of HuC32-2-72/CECA; SH3: sequential inoculation fermentation of HoC42/CECA.

## Data Availability

The original contributions presented in the study are included in the article, further inquiries can be directed to the corresponding authors.

## Appendix A

**Table A1:** 26S D1/D2 fragment sizes of non-Saccharomyces yeasts and their identities compared to the reference yeasts.

Strain	Size (bp)	Related Yeast	Type Strain	Identity (%)	Isolate Source
Hg01W23	629	*Hanseniaspora guilliermondii* TY20	FJ972220.1	99.67	Jingyang, Shaanxi
HgA-1-2	628	*Hanseniaspora guilliermondii* TY20	FJ972220.1	100.00	Jingyang, Shaanxi
HgA17-2-10	640	*Hanseniaspora guilliermondii* TY20	FJ972220.1	100.00	Shanshan, Xinjiang
HgB-1-4	634	*Hanseniaspora guilliermondii* TY20	FJ972220.1	99.50	Jingyang, Shaanxi
HgC-2-4	634	*Hanseniaspora guilliermondii* TY20	FJ972220.1	100.00	Jingyang, Shaanxi
HgC-3-1	633	*Hanseniaspora guilliermondii* TY20	FJ972220.1	99.83	Jingyang, Shaanxi
HgC-4-4	630	*Hanseniaspora guilliermondii* TY14	FJ972216.1	99.50	Jingyang, Shaanxi
HgD-1-1	635	*Hanseniaspora guilliermondii* TY20	FJ972220.1	99.18	Jingyang, Shaanxi
HgD-1-5	631	*Hanseniaspora guilliermondii* TY14	FJ972216.1	99.34	Jingyang, Shaanxi
Ho01W25	623	*Hanseniaspora opuntiae* HCM-NM44	MK101218.1	99.51	Jingyang, Shaanxi
Ho23W64	627	*Hanseniaspora opuntiae* 11-1139	MH465522.1	99.83	Jingyang, Shaanxi
Ho23W68	625	*Hanseniaspora opuntiae* HCM-NM44	MK101218.1	99.67	Jingyang, Shaanxi
HoA-1-3	618	*Hanseniaspora opuntiae* JEY269	KC111446.1	99.68	Jingyang, Shaanxi
HoB-1-1	633	*Hanseniaspora opuntiae* 11-1184	MH465493.1	99.35	Jingyang, Shaanxi
HoB-1-5	632	*Hanseniaspora opuntiae* JW11-4	KU316739.1	99.51	Jingyang, Shaanxi
HoB-2-1	629	*Hanseniaspora opuntiae* 11-1194	MH465395.1	99.83	Jingyang, Shaanxi
HoB-2-3	632	*Hanseniaspora opuntiae* HCM-NM44	MK101218.1	99.67	Jingyang, Shaanxi
HoB7-15	631	*Hanseniaspora opuntiae* JPMK66	MH118515.1	100.00	Jingyang, Shaanxi
HoC-1-3	633	*Hanseniaspora opuntiae* JW11-4	KU316739.1	99.67	Jingyang, Shaanxi
HoC-1-4	634	*Hanseniaspora opuntiae* SM10UFAM	MN268780.1	99.67	Jingyang, Shaanxi
HoC-1-5	636	*Hanseniaspora opuntiae* CEC 31C-9	KF263943.1	99.83	Jingyang, Shaanxi
HoC-3-3	631	*Hanseniaspora opuntiae* SM10UFAM	MN268780.1	100.00	Jingyang, Shaanxi
HoC-4-1	634	*Hanseniaspora opuntiae* 11-1139	MH465522.1	99.51	Jingyang, Shaanxi
HoC-4-2	633	*Hanseniaspora opuntiae* JEY269	KC111446.1	99.83	Jingyang, Shaanxi
HoD-1-2	635	*Hanseniaspora opuntiae* CEC 31C-9	KF263943.1	99.50	Jingyang, Shaanxi
HoD-1-3	630	*Hanseniaspora opuntiae* CEC 31C-9	KF263943.1	99.34	Jingyang, Shaanxi
Hu13W76	631	*Hanseniaspora uvarum* X21-10	MN337251.1	99.51	Jingyang, Shaanxi
HuA6-1-6	637	*Hanseniaspora uvarum* NS-O-182	KT922901.1	100.00	Shanshan, Xinjiang
HuA11-1-2	620	*Hanseniaspora uvarum* CEC 32SA-51	KF263942.1	99.35	Shanshan, Xinjiang
HuB1S-6	628	*Hanseniaspora uvarum* NS-PDC-10	KT922480.1	99.67	Jingyang, Shaanxi
HuB-2-2	634	*Hanseniaspora uvarum* CBS:2589	KY107852.1	99.67	Jingyang, Shaanxi
HuBG-1-5	633	*Hanseniaspora uvarum* NS-EM-77	KT922349.1	99.67	Jingyang, Shaanxi
HuBG-1-6	636	*Hanseniaspora uvarum* NS-O-241	KT922960.1	100.00	Jingyang, Shaanxi
HuC-2-3	632	*Hanseniaspora uvarum* 11-1148	MH465536.1	99.83	Jingyang, Shaanxi
HuC-2-5	635	*Hanseniaspora uvarum* HN-NM-67	MK101217.1	99.35	Jingyang, Shaanxi
HuC-3-2	629	*Hanseniaspora uvarum* HuW1	KF992155.1	99.51	Jingyang, Shaanxi
HuC-3-5	634	*Hanseniaspora uvarum* X21-10	MN337251.1	99.18	Jingyang, Shaanxi
HuC-4-5	631	*Hanseniaspora uvarum* X22-9	MN337255.1	99.67	Jingyang, Shaanxi
HuC413	633	*Hanseniaspora uvarum* NS-O-51	KT922774.1	99.83	Gansu Mogao Winery
HuC466	637	*Hanseniaspora uvarum* J15-5	MN337257.1	99.66	Gansu Mogao Winery
HuC534	639	*Hanseniaspora uvarum* NS-EM-122	KT922393.1	100.00	Gansu Mogao Winery
HuD-1-4	630	*Hanseniaspora uvarum* NS-O-51	KT922744.1	99.83	Jingyang, Shaanxi
HuD-2-1	623	*Hanseniaspora uvarum* J15-5	MN337257.1	99.51	Jingyang, Shaanxi
HuD-2-3	633	*Hanseniaspora uvarum* NS-O-16	KT922739.1	99.67	Jingyang, Shaanxi
HuD-2 -4	635	*Hanseniaspora uvarum* X21-10	MN337251.1	100.00	Jingyang, Shaanxi
HuM69	635	*Hanseniaspora uvarum* NS-EM-82	KT922353.1	100.00	Manas, Xinjiang
HuF-2-1	637	*Hanseniaspora uvarum* NS-O-241	KT922960.1	99.49	Ningxia Royal Horse Winery
MafA5-5-3	558	*Metschnikowia aff. fructicola* D3895	AM286804.1	99.81	Shanshan, Xinjiang
MafA9-4-5	578	*Metschnikowia aff. fructicola* C83	EU373448.1	99.80	Shanshan, Xinjiang
MafA11-1-1	629	*Metschnikowia aff. fructicola* D3895	AM286804.1	99.81	Shanshan, Xinjiang
MafA13-1-3	559	*Metschnikowia aff. fructicola* D3895	AM286804.1	99.81	Shanshan, Xinjiang
MafC83	555	*Metschnikowia aff. fructicola* D3895	AM286804.1	99.81	Gansu Mogao Winery
MafF-1-7	558	*Metschnikowia aff. fructicola* D3895	AM286804.1	99.62	Ningxia Royal Horse Winery
PklC734	616	*Pichia kluyveri* X27-5	MN337254.1	99.51	Gansu Mogao Winery
PklM114	617	*Pichia kluyveri* X23-10	MN337239.1	99.66	Manas, Xinjiang
PklM118	631	*Pichia kluyveri* X23-10	MN337239.1	99.66	Manas, Xinjiang
PklM144	625	*Pichia kluyveri* CBS:7274	KY108823.1	99.66	Manas, Xinjiang
PklFS-2-7	630	*Pichia kluyveri* YG14	MF045458.1	99.83	Ningxia Royal Horse Winery
PkuB4-19	625	*Pichia kudriavzevii* CK12	MN712334.1	99.66	Jingyang, Shaanxi
PkuB4S-1	616	*Pichia kudriavzevii* CBS:5147	MH545928.1	99.83	Jingyang, Shaanxi
PkuB6-1	619	*Pichia kudriavzevii* ZJ-13	KY283161.1	99.66	Jingyang, Shaanxi
PkuB9S-2	618	*Pichia kudriavzevii* CK12	MN712334.1	100.00	Jingyang, Shaanxi
PkuC392	617	*Pichia kudriavzevii* CK12	MN712334.1	99.17	Gansu Mogao Winery
PkuC396	570	*Pichia kudriavzevii* CK12	MN712334.1	100.00	Gansu Mogao Winery
PkuGS-1-18	622	*Pichia kudriavzevii* CK12	MN712334.1	100.00	Helan Mountain, Ningxia
PkuGS-1-20	621	*Pichia kudriavzevii* CK12	MN712334.1	99.83	Helan Mountain, Ningxia
PkuM-1-1	621	*Pichia kudriavzevii* CK12	MN712334.1	99.83	Helan Mountain, Ningxia
PkuM100	621	*Pichia kudriavzevii* 4_5	MF461004.1	100.00	Manas, Xinjiang
PkuM101	622	*Pichia kudriavzevii* 4_5	MF461004.1	100.00	Manas, Xinjiang
PkuM130	623	*Pichia kudriavzevii* CBS:5147	MH545928.1	100.00	Manas, Xinjiang
PkuM131	625	*Pichia kudriavzevii* CBS:5147	MH545928.1	100.00	Manas, Xinjiang

**Table A2:** Fermentation parameters and secondary metabolites of the 71 non-*Saccharomyces* strains and *S. cerevisiae* CECA in synthetic grape juice *.

Strain	OD_600nm_	Residual Sugar (g/L)	Sugar Consumption (g/L)	Ethanol (%*v*/*v*)	Ethanol Yield (g/g)	Changes in Ethanol Yield (%)	Glycerol (g/L)	Glycerol Yield (g/g)	Total Acid ^a^ (g/L)	Volatile Acid ^b^ (g/L)
*Saccharomyces cerevisiae*										
CECA ^c^	3.39 ± 0.04	1.82 ± 0.10	198.18 ± 0.10	10.16 ± 0.01	0.404 ± 0.000	0	5.30 ± 0.00	0.026 ± 0.000	7.31 ± 0.11	0.50 ± 0.01
*Hanseniaspora guilliermondii*										
Hg01W23	1.39 ± 0.13	110.04 ± 5.91	89.96 ± 5.91	3.25 ± 0.20	0.409 ± 0.014	−4.30	1.25 ± 0.07	0.014 ± 0.001	6.57 ± 0.47	0.52 ± 0.02
HgA-1-2	1.58 ± 0.25	104.17 ± 4.47	95.83 ± 4.47	3.70 ± 0.78	0.303 ± 0.020	−32.42	1.45 ± 0.05	0.015 ± 0.003	6.74 ± 0.16	0.49 ± 0.05
HgA17-2-10	2.21 ± 0.19	60.07 ± 2.94	139.93 ± 2.94	4.64 ± 0.51	0.261 ± 0.018	−40.11	2.50 ± 0.06	0.018 ± 0.003	6.06 ± 0.20	0.43 ± 0.03
HgB-1-4	1.10 ± 0.02	139.01 ± 1.10	60.99 ± 1.10	3.58 ± 0.37	0.465 ± 0.024	−1.96	3.10 ± 0.26	0.052 ± 0.002	6.22 ± 0.10	0.57 ± 0.01
HgC-2-4	1.24 ± 0.04	123.06 ± 2.46	76.94 ± 2.46	4.13 ± 0.67	0.425 ± 0.025	−13.94	3.03 ± 0.12	0.040 ± 0.006	6.69 ± 0.21	0.62 ± 0.05
HgC-3-1	1.22 ± 0.04	126.49 ± 1.39	73.51 ± 1.39	4.55 ± 0.30	0.489 ± 0.023	−1.04	2.97 ± 0.25	0.040 ± 0.001	6.62 ± 0.21	0.56 ± 0.03
HgC-4-4	1.12 ± 0.01	126.07 ± 3.33	73.93 ± 3.33	4.25 ± 0.27	0.455 ± 0.048	−8.01	2.90 ± 0.17	0.039 ± 0.001	6.55 ± 0.21	0.62 ± 0.02
*Hanseniaspora opuntiae*										
HoD-1-1	1.22 ± 0.02	131.10 ± 1.68	68.9 ± 1.68	4.03 ± 0.14	0.459 ± 0.015	−7.15	2.40 ± 0.17	0.035 ± 0.007	6.65 ± 0.18	0.53 ± 0.04
HoD-1-5	2.24 ± 0.13	15.08 ± 1.51	184.92 ± 1.51	9.49 ± 0.54	0.405 ± 0.027	−4.00	4.97 ± 0.09	0.027 ± 0.002	7.59 ± 0.18	0.82 ± 0.02
Ho01W25	1.83 ± 0.10	103.41 ± 6.99	96.59 ± 6.99	3.05 ± 0.40	0.252 ± 0.052	−43.82	1.07 ± 0.15	0.011 ± 0.002	6.01 ± 0.30	0.47 ± 0.02
Ho23W64	1.43 ± 0.11	118.63 ± 2.58	81.37 ± 2.58	3.53 ± 0.14	0.358 ± 0.015	−16.24	1.27 ± 0.15	0.016 ± 0.001	6.24 ± 0.25	0.46 ± 0.06
Ho23W68	1.63 ± 0.17	127.7 ± 3.58	72.30 ± 3.58	4.11 ± 0.21	0.494 ± 0.211	−0.03	1.77 ± 0.15	0.027 ± 0.003	6.58 ± 0.12	0.51 ± 0.04
HoA-1-3	2.46 ± 0.10	26.11 ± 2.13	173.89 ± 2.13	6.58 ± 0.39	0.299 ± 0.003	−30.98	3.25 ± 0.07	0.019 ± 0.002	6.27 ± 0.20	0.56 ± 0.07
HoB-1-1	1.54 ± 0.21	65.65 ± 2.06	134.35 ± 2.06	6.38 ± 0.41	0.378 ± 0.028	−13.35	3.13 ± 0.15	0.023 ± 0.004	7.12 ± 0.30	0.50 ± 0.08
HoB-1-5	1.15 ± 0.11	126.12 ± 3.75	73.88 ± 3.75	3.63 ± 0.20	0.389 ± 0.036	−21.38	3.23 ± 0.23	0.044 ± 0.001	6.32 ± 0.44	0.58 ± 0.06
HoB-2-1	1.17 ± 0.05	107.81 ± 3.72	92.19 ± 3.72	5.15 ± 0.26	0.445 ± 0.028	−0.84	2.43 ± 0.06	0.027 ± 0.003	6.85 ± 0.05	0.48 ± 0.07
HoB-2-3	1.70 ± 0.19	99.91 ± 2.40	100.09 ± 2.40	3.18 ± 0.32	0.250 ± 0.022	−44.17	1.33 ± 0.15	0.013 ± 0.001	6.54 ± 0.17	0.49 ± 0.03
HoB7-15	1.48 ± 0.08	124.65 ± 3.74	75.35 ± 3.74	4.26 ± 0.43	0.464 ± 0.006	−6.16	1.63 ± 0.15	0.023 ± 0.005	10.11 ± 0.39	1.02 ± 0.03
HoC-1-3	1.65 ± 0.11	116.74 ± 2.65	83.26 ± 2.65	2.96 ± 0.40	0.283 ± 0.008	−33.77	1.27 ± 0.21	0.015 ± 0.003	6.76 ± 0.28	0.47 ± 0.04
HoC-1-4	1.36 ± 0.17	103.72 ± 3.54	96.28 ± 3.54	5.61 ± 0.42	0.459 ± 0.018	2.28	2.83 ± 0.12	0.029 ± 0.003	6.48 ± 0.03	0.54 ± 0.02
HoC-1-5	1.23 ± 0.04	128.54 ± 1.01	71.46 ± 1.01	4.08 ± 0.61	0.469 ± 0.001	−5.11	2.80 ± 0.10	0.042 ± 0.005	6.38 ± 0.18	0.56 ± 0.04
HoC-3-3	1.23 ± 0.04	131.31 ± 1.65	68.69 ± 1.65	4.30 ± 0.47	0.492 ± 0.016	−0.43	3.27 ± 0.12	0.048 ± 0.008	6.85 ± 0.34	0.63 ± 0.04
HoC-4-1	1.10 ± 0.01	128.71 ± 2.77	71.29 ± 2.77	3.92 ± 0.39	0.434 ± 0.043	−12.21	2.73 ± 0.15	0.038 ± 0.003	6.46 ± 0.28	0.59 ± 0.03
HoC-4-2	2.40 ± 0.03	19.74 ± 1.65	180.26 ± 1.65	7.13 ± 0.70	0.314 ± 0.009	−25.66	4.97 ± 0.25	0.028 ± 0.001	7.44 ± 0.07	0.75 ± 0.03
HoD-1-2	1.24 ± 0.01	127.08 ± 2.47	72.92 ± 2.47	4.01 ± 0.23	0.435 ± 0.039	−12.00	2.87 ± 0.06	0.039 ± 0.001	6.52 ± 0.11	0.54 ± 0.03
HoD-1-3	1.89 ± 0.14	84.00 ± 1.89	116.00 ± 1.89	4.23 ± 0.47	0.301 ± 0.017	−21.53	1.75 ± 0.09	0.015 ± 0.002	5.98 ± 0.52	0.60 ± 0.03
*Hanseniaspora uvarum*										
Hu13W76	2.47 ± 0.19	69.44 ± 3.67	130.56 ± 3.67	4.12 ± 0.69	0.244 ± 0.002	−42.19	1.93 ± 0.17	0.014 ± 0.001	6.17 ± 0.50	0.67 ± 0.03
HuA6-1-6	1.85 ± 0.20	99.8 ± 5.47	100.2 ± 5.47	4.30 ± 0.18	0.339 ± 0.014	−24.39	1.93 ± 0.12	0.019 ± 0.000	6.05 ± 0.26	0.61 ± 0.02
HuA11-1-2	2.02 ± 0.14	43.85 ± 4.13	156.15 ± 4.13	3.66 ± 0.24	0.185 ± 0.005	−58.01	1.43 ± 0.09	0.009 ± 0.003	6.11 ± 0.58	0.55 ± 0.04
HuB1S-6	1.84 ± 0.26	106.31 ± 4.79	93.69 ± 4.79	3.03 ± 0.20	0.257 ± 0.004	−42.73	1.35 ± 0.07	0.014 ± 0.000	7.15 ± 0.05	0.44 ± 0.01
HuB-2-2	2.56 ± 0.09	2.46 ± 0.26	197.54 ± 0.26	7.72 ± 0.59	0.308 ± 0.001	−24.13	5.20 ± 0.17	0.028 ± 0.001	7.16 ± 0.09	0.85 ± 0.03
HuBG-1-5	2.46 ± 0.20	13.58 ± 1.28	186.42 ± 1.28	8.52 ± 0.04	0.360 ± 0.022	−12.49	4.90 ± 0.28	0.026 ± 0.002	7.73 ± 0.03d	0.81 ± 0.01
HuBG-1-6	2.15 ± 0.07	20.64 ± 1.55	179.36 ± 1.55	7.56 ± 0.35	0.332 ± 0.016	−21.52	4.60 ± 0.14	0.026 ± 0.002	7.62 ± 0.21	0.76 ± 0.04
HuC-2-3	2.50 ± 0.10	17.11 ± 1.64	182.89 ± 1.64	9.52 ± 0.26	0.411 ± 0.010	−2.73	4.90 ± 0.14	0.025 ± 0.001	7.52 ± 0.14	0.81 ± 0.03
HuC-2-5	1.89 ± 0.16	110.91 ± 1.72	89.09 ± 1.72	3.01 ± 0.38	0.271 ± 0.008	−36.59	1.20 ± 0.06	0.013 ± 0.002	6.76 ± 0.32	0.51 ± 0.05
HuC-3-2	2.32 ± 0.20	24.63 ± 0.68	175.37 ± 0.68	6.13 ± 0.29	0.274 ± 0.014	−33.67	5.43 ± 0.25	0.030 ± 0.001	7.19 ± 0.38	0.75 ± 0.10
HuC-3-5	2.31 ± 0.17	18.88 ± 1.07	181.12 ± 1.07	8.02 ± 0.07	0.349 ± 0.031	−17.39	3.80 ± 0.02	0.022 ± 0.003	7.69 ± 0.34	0.86 ± 0.03
HuC-4-5	1.47 ± 0.12	120.88 ± 4.31	79.12 ± 4.31	2.98 ± 0.38	0.307 ± 0.027	−28.03	1.00 ± 0.00	0.013 ± 0.000	6.88 ± 0.09	0.44 ± 0.06
HuC413	2.11 ± 0.15	21.87 ± 2.05	178.13 ± 2.05	7.63 ± 0.59	0.337 ± 0.065	−19.13	4.83 ± 0.31	0.027 ± 0.001	7.42 ± 0.12	0.87 ± 0.05
HuC466	0.97 ± 0.05	156.07 ± 2.93	43.93 ± 2.93	2.39 ± 0.26	0.429 ± 0.002	7.13	1.35 ± 0.07	0.036 ± 0.002	11.03 ± 0.73	0.90 ± 0.03
HuC534	2.16 ± 0.03	16.00 ± 0.43	184.00 ± 0.43	7.42 ± 0.15	0.317 ± 0.015	−24.86	4.90 ± 0.26	0.027 ± 0.002	7.44 ± 0.27	0.70 ± 0.04
HuD-1-4	1.01 ± 0.13	145.83 ± 1.83	54.17 ± 1.83	3.19 ± 0.25	0.464 ± 0.026	11.84	2.53 ± 0.23	0.047 ± 0.006	6.53 ± 0.21	0.55 ± 0.06
HuD-2-1	2.42 ± 0.17	7.34 ± 2.30	192.66 ± 2.30	10.47 ± 0.63	0.429 ± 0.021	6.18	5.07 ± 0.21	0.026 ± 0.001	7.41 ± 0.09	0.89 ± 0.03
HuD-2-3	1.29 ± 0.06	129.46 ± 1.65	70.54 ± 1.65	3.91 ± 0.37	0.441 ± 0.037	−10.77	2.63 ± 0.09	0.038 ± 0.002	6.72 ± 0.23	0.63 ± 0.00
HuD-2-4	2.34 ± 0.16	3.32 ± 0.62	196.68 ± 0.62	10.42 ± 0.41	0.418 ± 0.015	2.82	5.57 ± 0.38	0.028 ± 0.002	7.51 ± 0.34	1.00 ± 0.01
HuM69	1.66 ± 0.01	94.56 ± 4.31	105.44 ± 4.31	4.06 ± 0.24	0.305 ± 0.018	−26.42	1.55 ± 0.07	0.015 ± 0.001	7.28 ± 0.08	0.67 ± 0.06
HuF-2-1	2.61 ± 0.73	53.1 ± 1.58	146.90 ± 1.58	5.11 ± 0.45	0.276 ± 0.030	−34.81	2.53 ± 0.38	0.017 ± 0.002	6.16 ± 0.37	0.58 ± 0.01
*Pichia kluyveri*										
PklC734	2.09 ± 0.14	3.68 ± 0.12	196.32 ± 0.12	8.55 ± 0.10	0.343 ± 0.006	−15.39	6.90 ± 0.20	0.035 ± 0.001	8.00 ± 0.24	0.98 ± 0.04
PklM114	2.99 ± 0.15	14.7 ± 0.31	185.30 ± 0.31	6.61 ± 1.47	0.280 ± 0.005	−31.81	5.50 ± 0.49	0.029 ± 0.007	7.81 ± 0.15	0.94 ± 0.02
PklM118	0.96 ± 0.06	93.81 ± 1.76	81.37 ± 1.76	2.93 ± 0.05	0.284 ± 0.009	−31.47	1.65 ± 0.09	0.020 ± 0.007	7.49 ± 0.14	0.50 ± 0.07
PklM144	2.30 ± 0.14	14.94 ± 1.33	185.06 ± 1.33	6.70 ± 0.60	0.286 ± 0.027	−30.42	6.67 ± 0.15	0.036 ± 0.001	7.66 ± 0.13	0.87 ± 0.05
PklFS-2-7	0.88 ± 0.09	139.86 ± 1.04	60.14 ± 1.04	3.35 ± 0.14	0.449 ± 0.010	−5.47	2.40 ± 0.06	0.041 ± 0.001	10.01 ± 0.16	0.98 ± 0.04
*Pichia kudriavzevii*										
PkuB4-19	2.12 ± 0.22	6.50 ± 2.02	193.50 ± 2.02	9.43 ± 1.16	0.384 ± 0.044	−3.39	6.60 ± 0.06	0.034 ± 0.002	8.10 ± 0.21	0.65 ± 0.04
PkuB4S-1	2.14 ± 0.05	3.28 ± 0.07	196.72 ± 0.07	8.94 ± 0.22	0.359 ± 0.005	−11.75	5.93 ± 0.15	0.030 ± 0.000	7.34 ± 0.26	0.66 ± 0.02
PkuB6-1	1.99 ± 0.20	10.56 ± 0.70	189.44 ± 0.70	6.88 ± 0.37	0.287 ± 0.014	−29.27	5.97 ± 0.45	0.031 ± 0.002	8.53 ± 0.25	0.57 ± 0.02
PkuB9S-2	1.48 ± 0.09	36.42 ± 0.52	163.58 ± 0.52	6.11 ± 0.25	0.295 ± 0.020	−34.03	5.40 ± 0.44	0.033 ± 0.004	8.51 ± 0.45	0.51 ± 0.03
PkuC392	1.41 ± 0.21	37.7 ± 2.42	162.3 ± 2.42	6.62 ± 0.27	0.322 ± 0.033	−28.12	4.21 ± 0.20	0.026 ± 0.006	7.49 ± 0.18	0.48 ± 0.02
PkuC396	1.81 ± 0.18	36.38 ± 2.76	163.62 ± 2.76	5.32 ± 0.46	0.258 ± 0.023	−42.42	4.07 ± 0.25	0.025 ± 0.002	7.21 ± 0.16	0.31 ± 0.02
PkuGS-1-18	1.33 ± 0.09	66.07 ± 5.59	133.93 ± 5.59	6.83 ± 0.11	0.403 ± 0.018	−7.61	6.10 ± 0.10	0.046 ± 0.001	6.63 ± 0.15	0.30 ± 0.01
PkuGS-1-20	1.46 ± 0.11	57.02 ± 5.65	142.98 ± 5.65	6.71 ± 0.12	0.370 ± 0.008	−12.62	6.33 ± 0.35	0.044 ± 0.004	9.71 ± 0.18	0.56 ± 0.03
PkuM-1-1	1.59 ± 0.11	39.55 ± 1.21	160.45 ± 1.21	7.12 ± 0.37	0.351 ± 0.032	−20.39	6.53 ± 0.47	0.041 ± 0.004	9.03 ± 0.26	0.52 ± 0.04
PkuM100	1.64 ± 0.23	40.37 ± 2.44	159.63 ± 2.44	4.30 ± 0.14	0.213 ± 0.006	−51.83	3.03 ± 0.17	0.019 ± 0.001	6.33 ± 0.16	0.41 ± 0.03
PkuM101	1.62 ± 0.19	36.72 ± 3.82	163.28 ± 3.82	5.74 ± 0.30	0.277 ± 0.008	−38.02	4.37 ± 0.32	0.027 ± 0.001	7.29 ± 0.18	0.40 ± 0.07
PkuM130	1.56 ± 0.11	43.76 ± 2.99	156.24 ± 2.99	6.82 ± 0.33	0.344 ± 0.017	−22.02	5.87 ± 0.06	0.038 ± 0.000	9.77 ± 0.26	0.57 ± 0.06
PkuM131	1.72 ± 0.17	44.40 ± 1.12	155.60 ± 1.12	5.48 ± 0.44	0.279 ± 0.026	−36.80	3.30 ± 0.13	0.021 ± 0.002	7.23 ± 0.35	0.35 ± 0.04
*Metschnikowia aff. fructicola*										
MafA5-5-3	1.32 ± 0.15	149.3 ± 4.79	50.70 ± 4.79	2.97 ± 0.11	0.460 ± 0.021	10.72	2.60 ± 0.00	0.052 ± 0.005	6.66 ± 0.20	0.16 ± 0.02
MafA9-4-5	2.25 ± 0.01	7.02 ± 0.91	192.98 ± 0.91	8.62 ± 0.96	0.351 ± 0.028	−13.15	7.40 ± 0.36	0.038 ± 0.001	7.69 ± 0.22	0.60 ± 0.02
MafA11-1-1	2.72 ± 0.11	2.47 ± 0.76	197.53 ± 0.76	8.87 ± 1.21	0.354 ± 0.029	−12.75	7.20 ± 0.22	0.036 ± 0.003	7.31 ± 0.56	0.63 ± 0.03
MafA13-1-3	2.31 ± 0.11	4.38 ± 0.55	195.62 ± 0.55	8.88 ± 0.54	0.358 ± 0.017	−11.88	7.33 ± 0.25	0.037 ± 0.001	7.28 ± 0.40	0.64 ± 0.05
MafC83	1.51 ± 0.15	121.98 ± 2.42	78.02 ± 2.42	3.35 ± 0.31	0.342 ± 0.051	−30.93	1.37 ± 0.15	0.018 ± 0.003	7.36 ± 0.22	0.10 ± 0.03
MafF-1-7	2.53 ± 0.11	1.85 ± 0.15	198.15 ± 0.15	7.25 ± 0.37	0.289 ± 0.005	−28.64	7.60 ± 0.10	0.038 ± 0.000	7.63 ± 0.37	0.67 ± 0.02

* The initial sugar concentration of synthetic must was 200 g/L. ^a^ Expressed as tartaric acid. ^b^ Expressed as acetic acid. ^c^ *S. cerevisiae* CECA was used as the control.
